# The influence of new beta-adrenolytics nebivolol and carvedilol on intraocular pressure and iris blood flow in rabbits

**DOI:** 10.1007/s00417-014-2623-5

**Published:** 2014-04-05

**Authors:** Dorota Szumny, Adam Szeląg

**Affiliations:** Department of Pharmacology, Wroclaw Medical University, ul. Mikulicza-Radeckiego 2, PL 50-345 Wrocław, Poland

**Keywords:** Beta-adrenolytics, Nebivolol, Carvedilol, Glaucoma, Intraocular pressure, Blood flow

## Abstract

**Background:**

The aim of this study was to assess the influence of propranolol, nebivolol, and carvedilol on intraocular pressure and blood flow in vessels of rabbit’s (New Zealand White) eyeball.

**Methods:**

The study was carried out on New Zealand white rabbits. Intraocular pressure was measured with the applanation tonometer Möller–Wedel and Icare; blood flow was measured with Doppler Laser Blood Flow Monitor MBD3.

**Results:**

Following a single administration into a conjunctival sac, all drugs decreased intraocular pressure. Iris blood flow was decreased following administration of propranol, but increased by nebivolol and carvedilol. After single and repeated oral administration of nebivolol and carvedilol an IOP decrease was demonstrated, but with no effect of all applied doses on iris or retina/choroid blood flow.

**Conclusion:**

Studies performed on an animal model indicate that it is possible to reduce the intraocular pressure and increase ocular blood flow in humans, following topical administration of carvedilol and nebivolol. Confirmation of those results in clinical trials may lead to development of a new anti-glaucoma treatment. Further clinical studies of long-term nebivolol and carvedilol are recommended. They are necessary for evaluation of usefulness of those drugs for selected groups of patients, for example those with glaucoma and arterial hypertension.

## Introduction

Glaucoma is one of the most common neurodegenerative conditions in ophthalmology. Optic nerve damage in course of glaucoma is most often caused by excessive intraocular pressure. Ocular ischaemia caused by circulatory disorders, for example in the course of a long-term arterial hypertension and nocturnal arterial pressure drops, both spontaneous and caused by hypotensives, is considered an equally important cause of that multifactorial condition. Those data suggest that an optimal hypotensive drug for patients with hypertension and concomitant glaucoma should not only reduce the intraocular pressure, but also improve ocular micro-circulation. In search for that type of drug, some hope is associated with novel β-adrenolytics — carvedilol or nebivolol, agents improving microcirculation by blocking α-adrenergic receptors and by stimulating nitrogen oxide synthesis, respectively, and reducing intraocular pressure. Their microcirculation-improving effect distinguishes those two drugs from majority of β-adrenolytics, that generally deteriorate peripheral blood flow.

Aqueous is produced by the ciliary apparatus in the posterior eye chamber by active secretion of sodium ions and hydrocarbons, ultrafiltration, and passive diffusion. Active secretion ensures production of approximately 80–90 % of volume of aqueous. The produced fluid flows into the anterior chamber, and leaves the eyeball using one of three ways: mostly via the trabeculum of the so-called filtration angle contained between the iris and cornea, via the choroid–sclera pathway and through vessels of the iris [1].

A significant role in regulation of aqueous secretion is played by receptors of the sympathetic system. Alpha_1A_- and alpha_1B_-adrenergic receptors are located in the iris, ciliary body, pupil dilator muscle, and retina of albino rabbits. Beta-adrenergic receptors are located in the ciliary body epithelium, suprascleral vein walls, and papillary sphincter. In rabbit eye, pupillary sphincter β-adrenergic receptors prevail, whereas muscarin receptors prevail in primates. Beta receptors were also discovered in epithelium of conjunctiva and cornea in rabbit’s eye, as well as in epithelium of iris, lens, choroid, and external muscles of the eyeball, and in the retina — similarly to humans [2].

Vascular system is similar in the human and rabbit eyeball. It is composed of two systems of arteries: ciliary (supplying blood to the choroid) and retinal. They are interconnected [3]. Both systems separate from the ocular artery, originating in the internal carotid artery. The most significant role in regulation of function of the ocular vascular system is played by α- and β-adrenergic receptors.

The L-arginine-NO system plays a role in physiological and pathological processes of the eyeball, and also in regulation of intraocular pressure (IOP) and ocular blood flow. NO was observed to play a very important role in diastole of blood vessels in all parts of the vascular membrane (iris, ciliary body, choroid), and also in the retina and eye adnexa vessels in rabbits. NO participates also in regulation of blood flow through the optic disc [4].

A study involving local or intra-aqueous administration of various NO donors to eyeballs of healthy rabbits indicated that increased activity of the L-arginine/NO/cGMP system causes reduction of IOP comparable to the effect of commonly used anti-glaucoma medications [5].

Elevated intraocular pressure and abnormal circulation (low arterial blood pressure and vascular regulation disorders) are considered main risk factors of glaucoma [6].

Incidence of arterial hypertension and glaucoma increases with age. Therefore, in elderly people the risk of coexistence of glaucoma and arterial hypertension is high. In patients with arterial hypertension and iatrogenic nocturnal drops of arterial pressure, it was demonstrated that reduced volume of blood reaching the optic disc is associated with glaucoma-induced injury of the optic disc [7].

Studies indicated that increased loss of visual field caused by glaucoma is associated with excessive reduction of arterial blood pressure during sleep [8]. Nocturnal reduction of arterial pressure by as much as 20 % was also observed in as much as 92 % of glaucoma patients, and in only 8 % of healthy subjects [9]. A correlation between the open-angle glaucoma and existence of impaired blood flow in retinal and extrabulbar circulation was demonstrated in clinical trials [10]. Blood flow in optic nerve vessels in patients with primary open angle glaucoma is 29 % lower than in healthy subjects [11]. An effect of alterations of arterial pressure, nocturnal hypotony, vessel ageing on progression of open-angle glaucoma was confirmed [10].

In ophthalmology, in glaucoma therapy, a group of non-selective antagonists of β-adrenergic receptors is one of two main groups of drugs used for reduction of intraocular pressure.

The purpose of the study was to evaluate an effect of selected β-adrenergic receptor antagonists: propranolol, nebivolol, and carvedilol, presenting various mechanisms of action, on intraocular pressure and blood flow in rabbit’s ocular vessel.

## Materials and methods

### Animals

The study was carried out on New Zealand white rabbits, of both sexes, with average body weight of 2.5–3 kg. All measurements — of intraocular pressure and blood flow — were performed in conscious rabbits, standing calmly on top of a table, held delicately without head immobilisation.

### Study substances

Propranolol hydrochloride, racemic nebivolol, carvedilol, L-NMMA, isoproterenol were purchased from Sigma-Aldrich (Sigma-Aldrich, Poznań, Poland). Commercially available forms of drugs were also used: 10 % phenylephrine hydrochloride (Neosynephrine, Alcon, Warszawa, Poland), 0.5 % timolol maleinate (Oftensin, Polpharma, Warszawa, Poland) and 1 % atropine sulphate (Atropinum sulfuricum, Polfa Warszawa, Warszawa, Polska).

**Intraocular pressure** measurement with the applanation tonometer Möller-Wedel (IJ Möller Optische Werke GmbH, Wedel, Germany) was performed following corneal anaesthesia with Thilorbin drops (0.4 % oxybuprocaine hydrochloride and 0.8 % sodium fluorescein, Alcon). Measurement with the Icare-VET apparatus (Icare Finland, Helsinki, Finland) required no previous anaesthesia.

**Blood flow** in the iris and retina/choroid was measured with the Doppler Laser Blood Flow Monitor MBD3 (Moor Instruments, Axminster, UK). Following local anaesthesia of the cornea (0.5 % proxymetacaine, Alcaine, Alcon) the laser probe was placed directly on rabbit’s cornea. Laser beam was directed perpendicularly to the surface of the iris and retina. The measurement time was 50 sec.

The probe measurement of the retinal surface was based in ultrasound measurement of length of the eyeball: distance between the cornea and the lens (3 mm on average), between the lens and the retina (12.5 mm on average), with corneal thickness of approx. 1 mm and rabbit aqueous refraction coefficient of 1.335, and the optic fiber diameter of 0.25 mm. It is approximately 0.3 mm^2^.

In the first part of the experiment drugs were administered topically intra-conjunctivally, in the second part orally (Table 1.). Effect of reference agents: timolol and atropine, on intraocular pressure was verified in the study group of animals before the experiment.

**Table 1:** The scheme of applanation

Stages of applanation								
Stage 1. Intraconjunctival applanation								
Group	Right eye	Left eye	Interval	Right eye	Left eye	Interval	Right eye	Left eye
I	0.1 % P	0.9 % NaCl	2 weeks	0.5 % P	0.9 % NaCl	2 weeks	1 % P	0.9 % NaCl
II	0.1 % N	0.9 % NaCl	2 weeks	0.5 % N	0.9 % NaCl	2 weeks	1 % N	0.9 % NaCl
III	0.1 % K	0.9 % NaCl	2 weeks	0.5 % K	0.9 % NaCl	2 weeks	1 % K	0.9 % NaCl
Stage 2. Intraconjunctival applanation								
Group	Right eye				Left eye			
II	0.5 % L-NMMA + 0.5 % N				0.9 % NaCl			
III	0.5 % K + 10 %F				0.9 % NaCl			
Stage 3. Intraconjunctival applanation								
Group	Right eye				Left eye			
I	0.5 % P + 0.5 % I				0.9 % NaCl			
II	0.5 % N + 0.5 % I				0.9 % NaCl			
III	0.5 % K + 0.5 % I				0.9 % NaCl			
Stage 4. Orally single applanation								
Substance	Dose	Interval	Dose		Interval		Dose	
K	0.2 mg/kg b.w.	5 days	1 mg/kg b.w.		5 days		5 mg/kg b.w.	
N	0.1 mg/kg b.w.	5 days	0.5 mg/kg b.w.		5 days		2.5 mg/kg b.w.	
Stage 5. Orally repeated applanation								
Substance		The day of applanation						
		1st day*	2nd day	3rd day*	4th day	5th day*	6th day	7th day*
		dose						
K		1 mg/kg b.w.	1 mg/kg b.w.	1 mg/kg b.w.	1 mg/kg b.w.	1 mg/kg b.w.	1 mg/kg b.w.	–
N		0.5 mg/kg b.w.	0.5 mg/kg b.w.	0.5 mg/kg b.w.	0.5 mg/kg b.w.	0.5 mg/kg b.w.	0.5 mg/kg b.w.	–

* The measurements were performed on appointed days.

P — propranolol, N — nebivolol, K — carvedilol, L-NMMA — N^G^-monomethyl-L-arginin, F — phenylephrine, I — isoproterenol, NaCl — 0.9 % NaCl

### Topical administration

For the first part of the experiment animals were divided into three groups, 12 rabbits each: group 1 received propranolol, group 2 nebivolol, and group 3 carvedilol. Study substances were administered into the right eye. Control substance (normal saline) was administered into the left eye. Solution of each study substance in concentration appropriate for the stage of the experiment, and the 0.9 % sodium chloride were administered into the conjunctival sack in volume of 1 drop (50 μl), measured with an automated pipette. Intraocular pressure was measured in both eyes before single drug administration (normal saline in the control group) and 1, 2, 3, and 5 h later. Blood flow in iris and retina/choroidea was measured before drug administration or normal saline in the control group and 1 and 2 h after.

Study drugs were administered in solutions, concentration of 0.1 %, 0.5 %, or 1 %. Each group received a particular drug in increasing concentrations; group 1 received propranolol at 0.1 % (3.86 mmol/l) concentration, followed by 0.5 % (19.3 mmol/l), and 1 % (38.6 mmol/l). Groups 2 and 3 received nebivolol 0.1 % (2.4 mmol/l), 0.5 % (12.3 mmol/l), 1 % (24 mmol/l) and carvedilol 0.1 % (2.4 mmol/l), 0.5 % (12.3 mmol/l), 1 % (24 mmol/l) respectively, according to the same scheme. In all groups, drugs were administered in 2-week intervals. (Table 1, Stage 1) The dose administered in a single drop (50 μl) was 50 μg for the drug in 0.1 % solution, 250 μg for the 0.5 % solution, and 500 μg for the 1 % solution.

Next, in group 2 the 0.5 % L-NMMA, an NO synthase inhibitor, was administered into the right ey, and after 10 min 0.5 % nebivolol. In group 3, 0.5 % carvedilol was administered into the right eye, and after 10 min phenylephrine, an α_1_ receptors agonist, into the same eye. (Table 1, Stage 2)

Next, nebivol was administered into the right eye in group 1, carvedilol in group 2, and in group 3 propranolol, and after 10 min 0.5 % isoproterenol, a β_1_ i β_2_ agonist. (Table 1, Stage 3)

### Oral administration

In the second part of the experiment, carvedilol and nebivolol were single administered orally to six rabbits. Another six rabbits were a control group. A single measurement of blood flow in the iris and retina/choroid, and of intraocular pressure was performed in both study and control groups. Measurements were completed 2 h after administration of carvedilol, and 3 h after administration of nebivolol. Carvedilol was administered in a dose of 0.2 mg/kg b.w. (0.4 mmol/kg b.w.) in 0.9 % NaCl, volume of 2 ml/kg b.w., followed by 1 mg/kg b.w. (2 mmol/kg b.w.) and 5 mg/kg b.w. (10 mmol/kg b.w.). Nebivolol was administered in a dose of 0.1 mg/kg b.w. (0.2 mmol/kg b.w.) in 0.9 % NaCl, volume of 2 ml/kg b.w., followed by 0.5 mg/kg b.w. (1 mmol/kg b.w.), and 2.5 mg/kg b.w. (5 mmol/kg b.w.). Animals in the control group received normal saline orally in the volume of 2 ml/kg b.w. Five-day intervals were maintained between subsequent tests. (Table 1, Stage 4)

Nebivolol and carvedilol were also administered in multiple doses orall. For the first 6 days, nebivolol was administered orally at a dose of 0.5 mg/kg b.w.(2 mmol/kg b.w.) in 0.9 % NaCl, volume of 2 ml/kg b.w. to six rabbits. Another six rabbits were the control group. Single measurements of blood flow in the iris and of intraocular pressure were performed on every other day (days 1, 3, 5, and 7) in the study group and the control. Seven days later, carvedilol at a dose of 1 mg/kg b.w. (1 mmol/kg b.w.) in 0.9 % NaCl, volume 2 ml/kg b.w, was administered in the same manner. Animals in the control group received 2 ml/kg b.w. of 0.9 % NaCl solution orally. (Table 1, Stage 5)

**Statistical analysis** utilised the ANOVA test, the Levene’s test, Student’s *t*-test and Tukey’s test (*p*-value below the threshold value 0.05). Statistica v. 9.0 was used for all of the statistical calculations. Before statistical analysis, the results were transformed to a normal distribution and an analysis of variance was carried out. The work includes the result already in the units in which the research was conducted.

We declare that the work was carried out according to statutory bioethical standards, and was approved by a bioethical committee (I Local Bioethical Committee in Wrocław, Poland: 6/2011, 14/2012 and 24/2012).

## Results

**Fig. 1:**
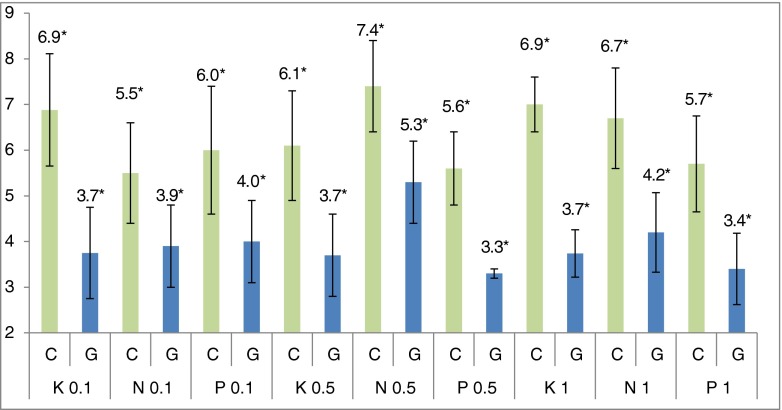
Mean values (*n* = 12) of intraocular pressure (in mmHg) measured 3 h after a single intraconjunctival administration of one drop of carvedilol (*K*), nebivolol (*N*) and propranolol (*P*). *Statistically significant difference between control (*C*) and study (*G*) groups

Our study demonstrated that propranolol, nebivolol, and carvedilol, following their topical administration into the conjunctival sack, at concentrations of 0.1 %, 0.5 % and 1 %, reduce intraocular pressure. Figure 1 presents the result of IOP measurement 3 hours after administration, when the lowest mean pressure values were observed. The values of IOP in the right eye (G) were compared to the left eye of the control group (C). Measurement was performed with the Möller–Wedel applanation tonometer. Blood-flow reduction in the iris was also demonstrated following a single administration of propranolol solution into the conjunctival sack, and increase of the flow following single doses of nebivolol and carvedilol (Tables 2 and 3). None of the studied β-adrenolytics affected vascular flow in the retina/choroid, following a single administration. Results of studies on receptor agonists and antagonists were not included in this paper, considering its limited volume.

**Table 2:** Differences between mean iris blood flow values

	P 0.1 %	P 0.5 %	P 1 %	N 0.1 %	N 0.5 %	N 1 %	K 0.1 %	K 0.5 %	K 1 %
1 h	**−233**	**−253**	**−247**	**123**	**107**	**198**	**98**	**127**	**142**
2 h	−104	−106	−84	60	60	**205**	**86**	**99**	**121**

Written in **bold** — statistically significant change of iris blood flow (positive — increasing, negative — decreasing of iris blood flow value)

**Table 3:** The presence of statistically significant differences of iris blood flow value between study and control group in 1 h and 2 h after a single intraconjunctival administration of propranolol, nebivolol, carvedilol

	P 0.1 %	P 0.5 %	P 1 %	N 0.1 %	N 0.5 %	N 1 %	K 0.1 %	K 0.5 %	K 1 %
1 h	↓	↓	↓	↑	↑	↑	↑	↑	↑
2 h	−	−	−	−	−	↑	↑	↑	↑

− no statistically significant differences; ↓ statistically significant decrease iris blood flow; ↑ statistically significant increase iris blood flow

**Fig. 2:**
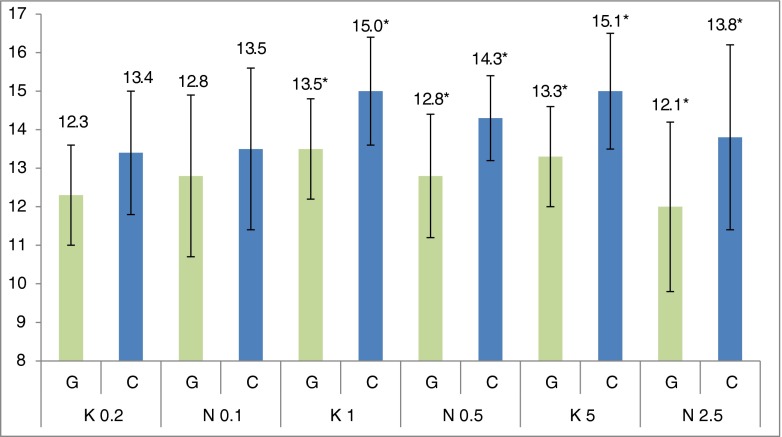
Mean values (*n* = 12) of intraocular pressure (in mmHg) following a single oral administration of carvedilol in 0.2, 1, and 5 mg/kg b.w. doses, and nebivolol in 0.1, 0.5, and 2.5 mg/kg b.w. doses in NaCl solution, volume of 2 ml/kg b.w. *Statistically significant difference between control (*C*) and study (*G*) groups

Following a single oral administration of nebivolol (N) and carvedilol (K), reduced IOP was demonstrated for both drugs and all applied doses, and no effect on the vascular flow in the iris, retina, or choroid (Fig. 2). The IOP values of the study group (G) were compared to the control group (C). The control group represented the IOP in both eyes before oral administration of the drug. Measurement was performed with the IcareVET tonometer.

Following a single, oral administration of carvedilol and nebivolol, a tendency was observed for reduction of mean values of vascular flow in the iris and retina/choroid in study groups, but differences between study groups and the control was not statistically significant.

**Fig. 3:**
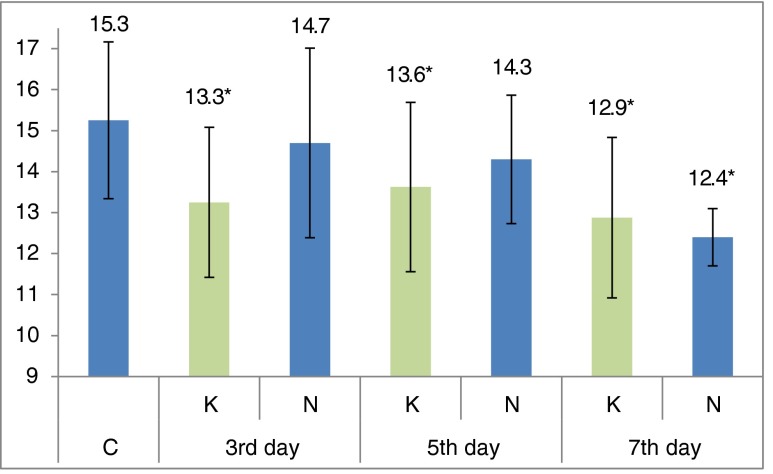
Mean values (*n* = 12) of intraocular pressure (in mmHg) measured in both eyes at 3, 5, and 7 days in a group which everyday received carvedilol (*K*) and nebivolol (*N*). *Statistically significant difference between control (*C*) and study (*K* or *N*) groups

Results obtained following multiple orally administration demonstrated a reduction of mean IOP after carvedilol and nebivolol administration, and no effect of both drugs on vascular flow in the iris and retina/choroid (Fig. 3). The IOP values of study group (G) were compared to the control group (C). The control group represented the IOP in both eyes measured on the first day of the experiment (pre-treatment). Measurement was performed with the IcareVET tonometer. Statistically significant differences between mean IOP values in the study group and the control group were observed for carvedilol on days 3, 5, and 7 of the experiment, and only on day 7 of the experiment for nebivolol. That may suggest a more rapid IOP-reducing effect of carvedilol by its influence on α-adrenergic receptors, compared to nebivolol acting on synthesis of nitrogen oxide.

Following a multiple oral dose, a tendency for initial reduction and subsequent increase of mean blood flow values in the iris was observed for carvedilol, and no effect on blood flow was observed for nebivolol. However, the difference between mean vascular flow values in the carvedilol group and the control was not statistically significant. Those results may be considered in planning of further clinical studies on the effect of carvedilol on ocular vascular flow following multiple oral administration.

Intraocular pressure was measured with various tonometers, obtaining various mean values. In the case of the applanation tonometer those values are approx. 6 mmHg, in case of the Icare tonometer approx. 9 mmHg, and in the case of the Icare-VET tonometer approx. 14 mmHg.

## Discussion

The search for drugs that could not only reduce intraocular pressure, but also have some additional properties, for example: a favorable effect on vascular flow, is a long one. Nipradilol - β- and α_1_-adrenolytic and NO donor, used for glaucoma therapy in Japan, may be an example. It has been demonstrated that the drug causes diastole of retinal blood vessels. Researchers believe that this effect is a result of both α_1_-adrenolitic activity and of nitrous oxide released following administration of nipradilol [12].

Nebivolol and carvedilol are both drugs commonly used in therapy of arterial hypertension, having a diastolic effect on blood vessels. However, available literature provides no information about a possible effect of nebivolol and carvedilol on ocular blood flow. Their diastolic effect on microcirculation vessels beyond the eyeball is well-documented.

Dog studies have allowed observation of coronary vessels diastole with nebivolol. That effect was eliminated by previous administration of NO synthase inhibitor (L-NMMA) [13]. Those observations have been confirmed in humans. Other authors have studied effects of chronic nebivolol and atenolol administration on function of epithelium and NO release. Intensification of the vasodilating effect was found following administration of acetylcholine in patients on nebivolol, contrary to patients on atenolol. A weaker L-NMMA (nitrous oxide synthase inhibitor) vasoconstricting effect was demonstrated in the group receiving nebivolol (14).

Experiments carried out on animals and on a group of patients with freshly diagnosed arterial hypertension confirmed the superiority of nebivolol in relation to the risk of impotence as an side-effect of hypotensive therapy, compared to older β-adrenolytics, such as atenolol and metoprolol [14, 15].

An effect of the nitroergic system on regulation of ocular blood flow has been studied recently. A role of that system in maintenance of vascular tension and in NO-mediated vasodilating effect in the eyeball had been confirmed previously in animal studies, including rabbits, and in humans [16].

Some researchers had previously been of the opinion that NO-releasing drugs or compounds are contraindicated in glaucoma. Reduced IOP following a systemic administration of NOS (methyl ester of N-nitro-L-arginine) inhibitors, caused by systole of ciliary body vessels in rabbits has been demonstrated (17). More recent studies indicate that increased NO concentration causes reduction of IOP comparable to that caused by standard anti-glaucoma medications [4, 5, 17].

Carvedilol increases epithelium-dependent diastole of coronary vessels, reduces vascular resistance, causes an epithelium-dependent diastole of arterioles, and improves flow in coronary microcirculation [18].

Beta-adrenolytics possessing an additional vasidilating effect, such as nebivolol or carvedilol, are also recommended for therapy of peripheral vascular conditions, including obliterative atheromatosis of lower limbs or Reynaud’s syndrome.

Completed studies of orally administered drugs may suggest that for patients with arterial hypertension and concomitant glaucoma associated with increased IOP, carvedilol or nebivolol may be favourable, because those drugs reduce not only the systemic blood pressure but also intraocular blood pressure.

That difference in IOP, measured by different tonometers, is a result of structural differences between rabbit and human eye, and of the fact that the Icare-VET tonometer was specifically designed for measurements in animals — and differences between rabbit and human eyes were accounted for in its case. These observations are consistent with a few scientific reports of significantly higher IOP values. These differences are explained by higher (compared to applanation tonometers) effect of corneal thickness on the result of a measurement with the Icare method [19].

Experiments demonstrated usefulness of Doppler Laser Blood Flow Monitor MBD3 for studies on rabbits’ eyes. According to the available bibliography and information obtained from the manufacturer, studies of retinal and choroidal vascular flow are innovative, and no one has attempted them before. The equipment has been used for examination of blood flow in the posterior segment of an eye in rats only [20].

As far as we know, this is the first study of nebivolol and carvedilol in the context of their possible use in therapy of glaucoma.

Obtained results suggest necessity of further clinical trials on nebivolol and carvedilol effect on IOP and blood flow following topical administration into the conjunctival sack. The additional improvement of blood flow is a favorable effect, because it targets the second (after increased IOP) cause of glaucoma — reduced vascular flow.
